# A Bad Beginning

**Published:** 1910-11-12

**Authors:** 


### The Hospital

A Journal of

The Medical Sciences and Hospital Administration.

New Series. No. 196, Vol. VIII. [No. 1268, Vol. XLIX.] SATURDAY, NOVEMBER 12 1910.

### A Bad Beginning.

There are few things which a certain section of the Press loves better than to attack some hospital or other, usually on a garbled version of occurrences narrated by prejudiced or ignorant people. A flaming headline, worded to suit the tastes of a public who unfortunately prefers to be amused rather than to be told the truth, is a certain "draw" with the class of readers for whom these purveyors of scandal cater. An example of this increasing, but regrettable, tendency has quite recently been furnished in the first issue of a new weekly paper, which starts with high-sounding professions and expressly declines to "pander to morbid sensationalism." In this particular case the institution attacked is the Great Ormond Street Hospital for Sick Children, which, as everyone knows, is the largest children's hospital in London. Moreover, it is a hospital which is always struggling hard against financial embarrassments, due to the enormous amount of work which is carried on there and to its scanty endowments. It lives from hand to mouth perpetually, and is particularly likely to suffer pecuniary loss from any imputation against the efficiency of its management. These facts, it may be urged, should render it especially sensitive to the merest breath of reproach; and so, in fact, it is. Need it be added that to drag the name of such a society needlessly in the mud is an act which every right-minded citizen should condemn?

The facts of the case, as alleged in the periodical, are few. We have taken no steps to ascertain from the hospital what other version of these facts there may be, because, even if they are as stated, they afford no justifiable ground, in our belief, for the course which has been taken. It is said that two female children were discharged about the beginning of March from the convalescent home at Cromwell House, Highgate, which is managed in conjunction with the hospital; that when sent home the heads of both were found to be "dirty," and that both suffered from a "discharge." These euphemisms may be taken to mean that they were infested with lice and were infected with gonococci. Both children were readmitted to the hospital, and both are there still. Now if these facts are true they certainly prove that nearly eight months ago a deplorable laxity at the convalescent home led to two very serious incidents. At the same time it is noticeable that no further cases of the same sort have been brought to light, and therefore it may reasonably be assumed that due precautions have months ago been taken to prevent the recurrence of such infection. It is self-evident that anyone who is genuinely concerned to see remedied an abuse of this sort would do best to approach the committee through the Secretary; and that to rake up an eight months' old error of somebody's merely for the purpose of an effective journalistic horror is essentially to "pander to morbid sensationalism" as well as to inflict possible damage upon the hospital.

The editor did, it is true, send a representative to inquire. The secretary of the hospital, after seeing this person, immediately communicated with the Vice-Chairman, who granted an interview to the "commissioner"; the senior physician and the senior surgeon attended at the same time. But not content with the prospect of such an investigation, the reporter also saw the house surgeon, who very properly said but little and refused to produce the medical history sheets of the patients. These sheets are, of course, the property of the hospital, and are confidential; to show one to an outsider is a very wrong thing to do in any circumstances. The house surgeon is forthwith branded as "young and inexperienced"; it seems rather as if he behaved with a discretion which points to old acquaintance with the methods of the sensational Press. Let it not be forgotten that the house surgeons at this hospital are elected for six months only, and that, therefore, this officer cannot possibly have had anything to do with the original cases; and also that it is practically out of the question to get this appointment at all (to which the munificent reward of £20 is attached) without first having been house surgeon to a general hospital of high standing.

The "commissioner" was assured that due precautions are taken to prevent the spread of every sort of infection at Great Ormond Street; yet he professes to be "far from satisfied," though he does not indicate what further measures he thinks advisable. The child from whom the contagion spread was isolated as soon as its condition was noticed, though by that time the mischief was done; this the "commissioner" declares to be an "amazing" statement, though why it should be so regarded passes comprehension. As long as to err is human, so long will accidents happen in the best regulated hospitals. To pursue such error with public recriminations eight months after its commission, in headlines about "shocking disclosures," "foul disease," "lame excuse," and so forth, is a bad beginning. Such a self-appointed censor cannot be allowed to pose as a single-minded altruist, but labels himself with the trade-mark of those who live by exploiting the public taste for unsavouriness.

